# Supplementary material for: Uncertainty quantification in multivariable regression for material property prediction with Bayesian neural networks
Source: Sci Rep. 2024 May 8;14:10543. doi: 10.1038/s41598-024-61189-x (PMC11078957; doi:10.1038/s41598-024-61189-x)
Supplement: Supplementary file 1 — Supplementary Figures. [file 41598_2024_61189_MOESM1_ESM.docx]

**Supplementary Material**

**Uncertainty Quantification in Multivariable Regression for Material Property Prediction with Bayesian Neural Networks**

*Longze Li, Jiang Chang, Aleksandar Vakanski, Yachun Wang, Tiankai Yao, Min Xian*

Figures S.1 to S.3 present the histograms of the input features for the three datasets.

*
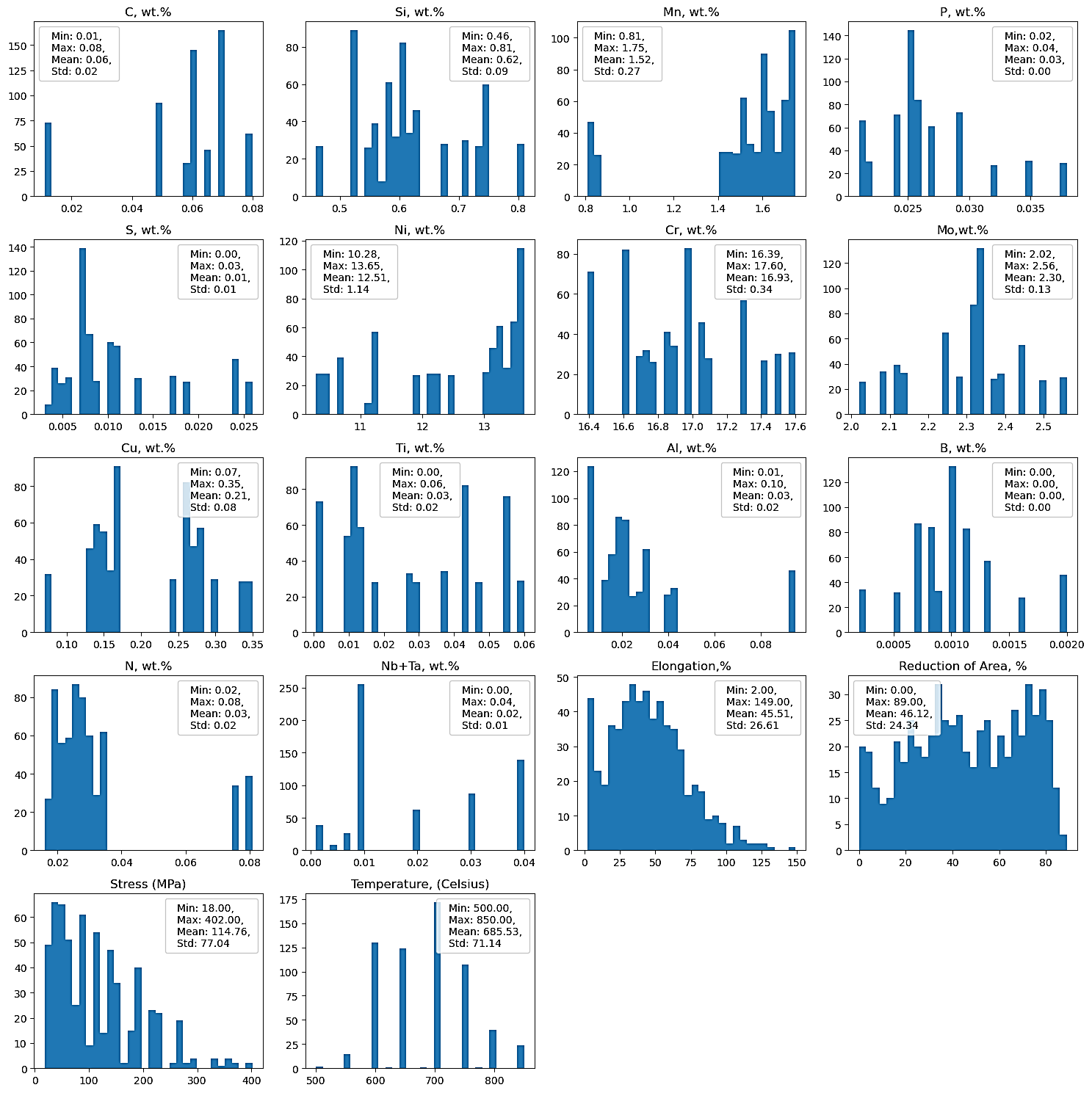
* ***Figure S.1*.** *Histograms of the input features for the stainless steel SS316 alloys dataset. “Min”, “Max”, “Mean”, and “Std” represent the minimum, maximum, mean, and standard deviation values, respectively.*

*
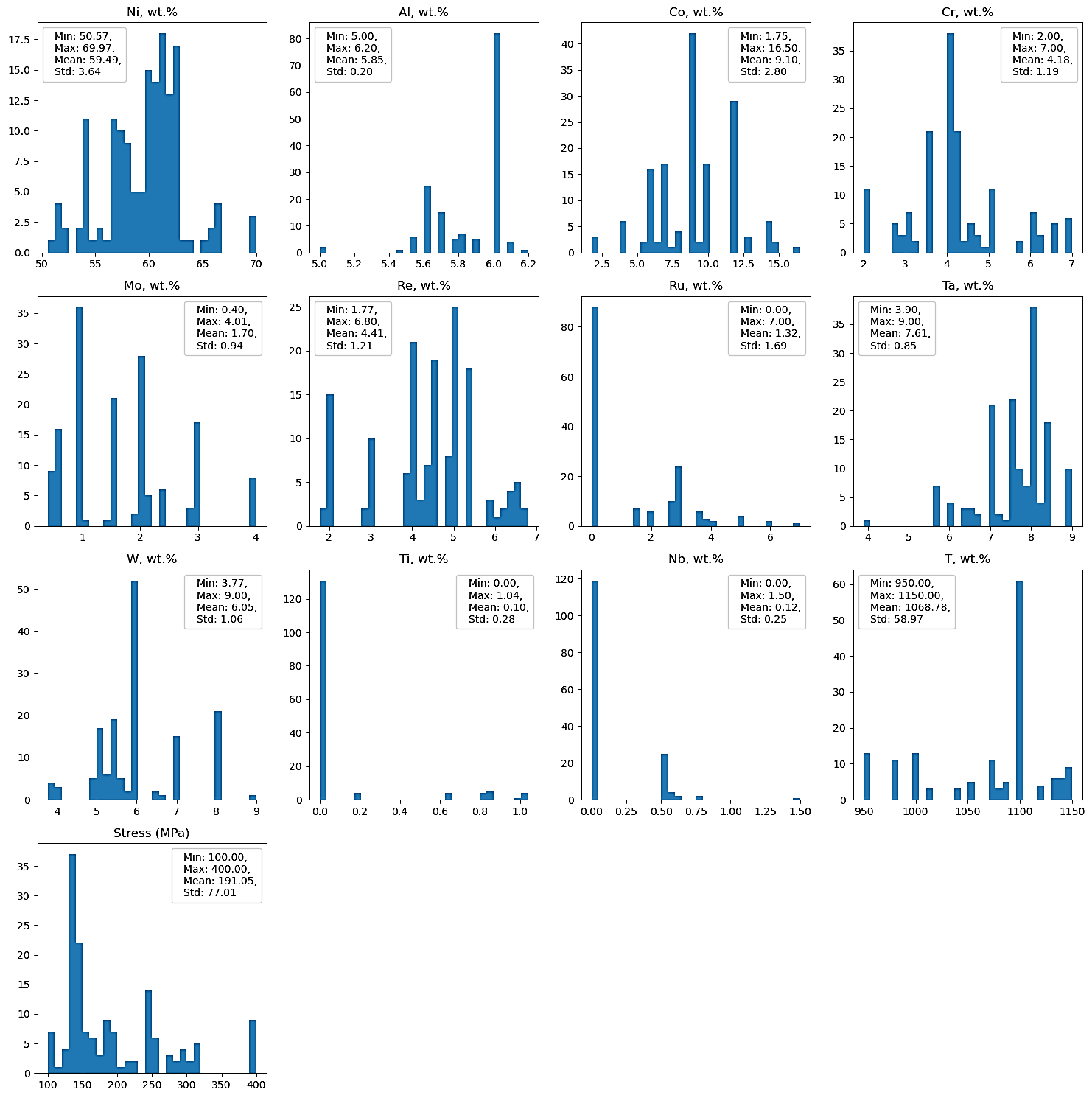
*

***Figure S.2*.** *Histograms of the input features for the Nickel-based superalloys dataset. “Min”, “Max”, “Mean”, and “Std” represent the minimum, maximum, mean, and standard deviation values, respectively.*

*
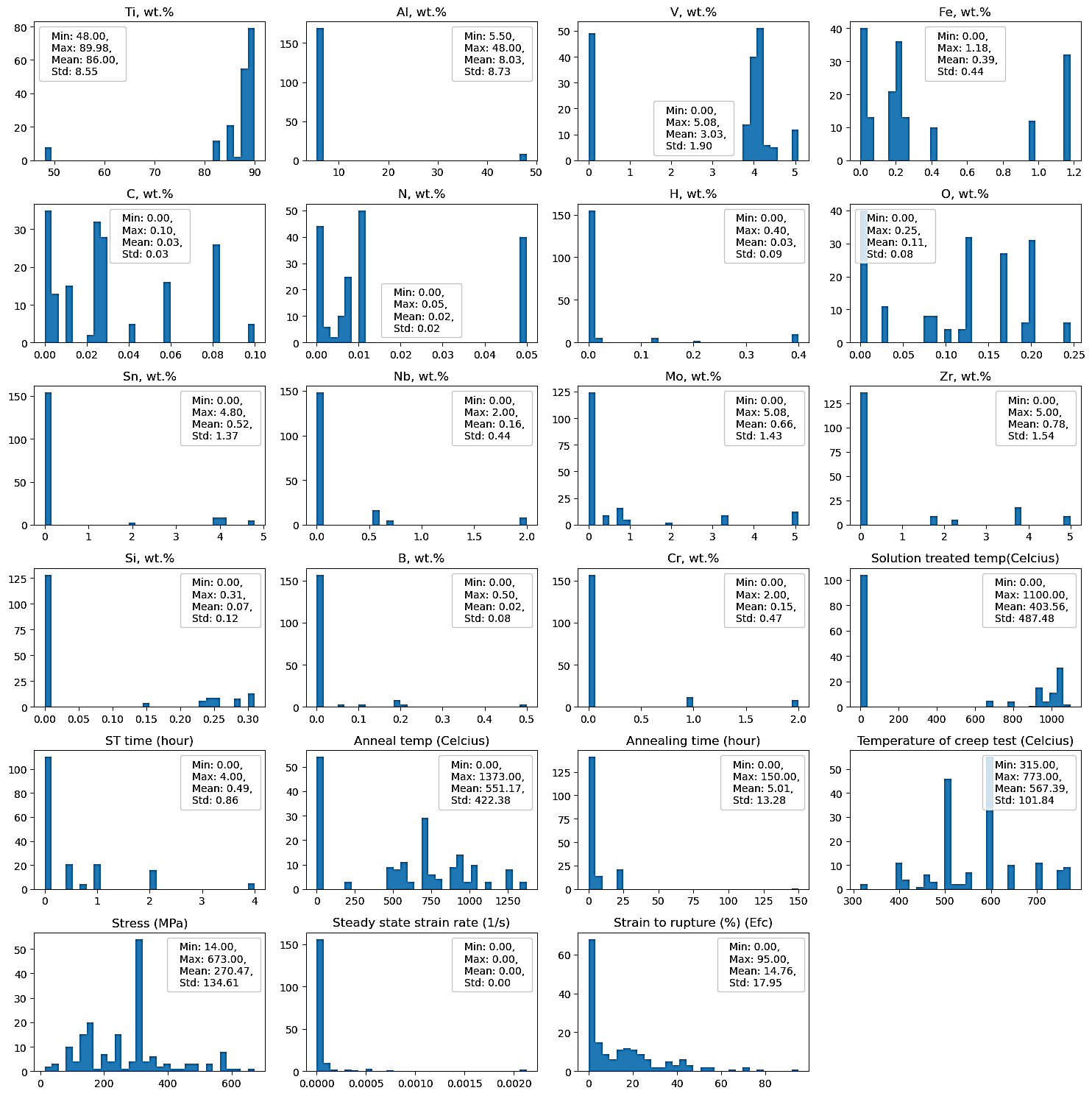
*

***Figure S.3*.** *Histograms of the input features for the Titanium-based alloys dataset. “Min”, “Max”, “Mean”, and “Std” represent the minimum, maximum, mean, and standard deviation values, respectively.*
